# Use of a wearable device to improve sleep quality

**DOI:** 10.3389/fdgth.2024.1384173

**Published:** 2025-02-04

**Authors:** Susan L. Moore, Evan P. Carey, Kristyna Finikiotis, Kelsey L. Ford, Richard D. Zane, Katherine K. Green

**Affiliations:** ^1^ mHealth Impact Lab, Department of Community and Behavioral Health, Colorado School of Public Health, University of Colorado Anschutz Medical Campus, Aurora, CO, United States; ^2^ Division of General Internal Medicine, University of Colorado School of Medicine, University of Colorado Anschutz Medical Campus, Aurora, CO, United States; ^3^ Department of Biostatistics & Informatics, Colorado School of Public Health, University of Colorado Anschutz Medical Campus, Aurora, CO, United States; ^4^ Care Access, Westminster, CO, United States; ^5^ Department of Emergency Medicine, University of Colorado School of Medicine, University of Colorado Anschutz Medical Campus, Aurora, CO, United States; ^6^ UCHealth CARE Innovation Center, University of Colorado Anschutz Medical Campus, Aurora, CO, United States; ^7^ Department of Otolaryngology, University of Colorado School of Medicine, University of Colorado Anschutz Medical Campus, Aurora, CO, United States

**Keywords:** sleep, sleep onset latency, sleep quality, sleep hygiene, health promotion, digital health

## Abstract

**Objectives:**

The present study aimed to analyze the effects of the use of a digital wellness device on improving sleep through reducing environmental noise.

**Methods:**

Fifty-five self-reported light or moderate sleepers with difficulty falling or staying asleep due to environmental noise participated in the study. Objective sleep architecture data were collected via a wireless electroencephalogram (EEG) sleep monitor and subjective data were obtained through analysis of daily sleep diaries and responses to study-specific user experience surveys. Four primary outcomes specified *a priori* were analyzed for statistical significance: objectively measured sleep onset latency (SOL), wake after sleep onset (WASO), number of awakenings, and perceived SOL. Exploratory analysis through descriptive statistics was conducted for an additional 36 secondary outcomes.

**Results:**

Use of the digital wellness device was associated with reduced SOL both objectively and subjectively. Perceived SOL was 32.5% reduced (*p* < 0.001, difference in means 7.5 min, 95% CI 22.3%–41.4% faster), and objectively measured SOL was 13.3% reduced (*p* = 0.030, difference in means 2.7 min, 95% CI = 1.4%–23.8% faster). No statistically significant differences were found for other primary outcomes. Among the subjective secondary outcomes, 100% of participants felt the device blocked environmental noise, 86% reported falling asleep more easily, 76% felt they stayed asleep longer, and 82% felt overall sleep quality was improved. No differences were observed among objectively measured secondary outcomes.

**Conclusions:**

Participants fell asleep faster when using the wearable wellness device. Participants also perceived sleep quality improvements with the intervention, although no objective differences were measured. These findings show promise for using noise-masking digital wellness devices in noisy environments to improve sleep quality.

## Introduction

The impact of good sleep on individual and population health is undeniable. Sufficient sleep is associated with a reduced risk of mortality (1–5), and sleep loss is associated with poorer quality of life, a reduced ability to function during daytime, increased healthcare utilization, and increased risk for conditions such as hypertension (2, 6–8), cerebrovascular disease (4, 5), diabetes (7, 9, 10), obesity (11–13), and depression (1, 14–16). Difficulties in sleep initiation and sleep maintenance have been shown to contribute to overall reduction in sleep quality (17). Trouble falling asleep at the beginning of the night or other intended sleep period is characteristic of sleep onset insomnia, where waking up during the night and having difficulty returning to sleep represents sleep maintenance insomnia, both of which are associated with reduced total sleep time, increased daytime sleepiness, reduced daytime functioning, and poorer overall sleep quality (17, 18).

Environmental noise has been shown to contribute to issues with sleep onset and sleep maintenance, significantly reduce sleep quality, and increase sleep disturbance (14, 19–21). Moreover, sleep disturbances and impaired sleep quality due specifically to environmental noise are associated with increased stress response, cardiometabolic dysfunction, and negative changes to both sleep architecture and perceived sleep quality (22). Exposure to environmental noise that impacts sleep, especially nighttime noise, is included in the rationale for defining noise as a public health hazard (23–26).

Many non-pharmacological interventions have been used to improve sleep quality by targeting sleep onset and sleep maintenance; however, the diversity of such non-pharmacological sleep-related interventions is vast and thus limits robust evaluation of efficacy across solutions (27). Examples with potential for overcoming delayed sleep onset and difficulties with sleep maintenance due to environmental noise include music-assisted relaxation, cognitive behavioral therapy, and relaxation training techniques (28–31). Other common solutions targeted toward reducing the impact of environmental noise on sleep quality include noise blocking (e.g., earplugs) and noise masking (e.g., white noise or soothing sounds), in which generated sound is used to affect the perception and experience of other sound (32–34). Commercial earbuds that are marketed towards improving sleep by masking sounds and relaxation techniques have been shown to improve sleep quality among health care providers (35, 36). However, limited evidence exists regarding the effectiveness of these technologies at improving sleep quality in the context of environmental noise in a wider population.

In this pre/post interventional pilot study, we sought to assess the efficacy of a commercial digital wellness product, the first-generation Bose noise-masking sleepbuds™ (Bose Corporation, Framingham, MA) at improving objectively measured and subjectively reported sleep quality for people with self-reported difficulty sleeping due to environmentally induced auditory disruption. We hypothesized using the device would improve sleep quality associated with improvements in sleep initiation and sleep maintenance. We assessed solution effect on sleep quality through measuring changes in objective and subjective sleep onset latency (SOL), wake after sleep onset (WASO), and number of awakenings, based on American Academy of Sleep Medicine workgroup diagnostic criteria for primary insomnia and domains for measuring sleep quality used in the Pittsburgh Sleep Quality Index (17, 37).

## Materials and methods

### Participants

**Figure 1:**
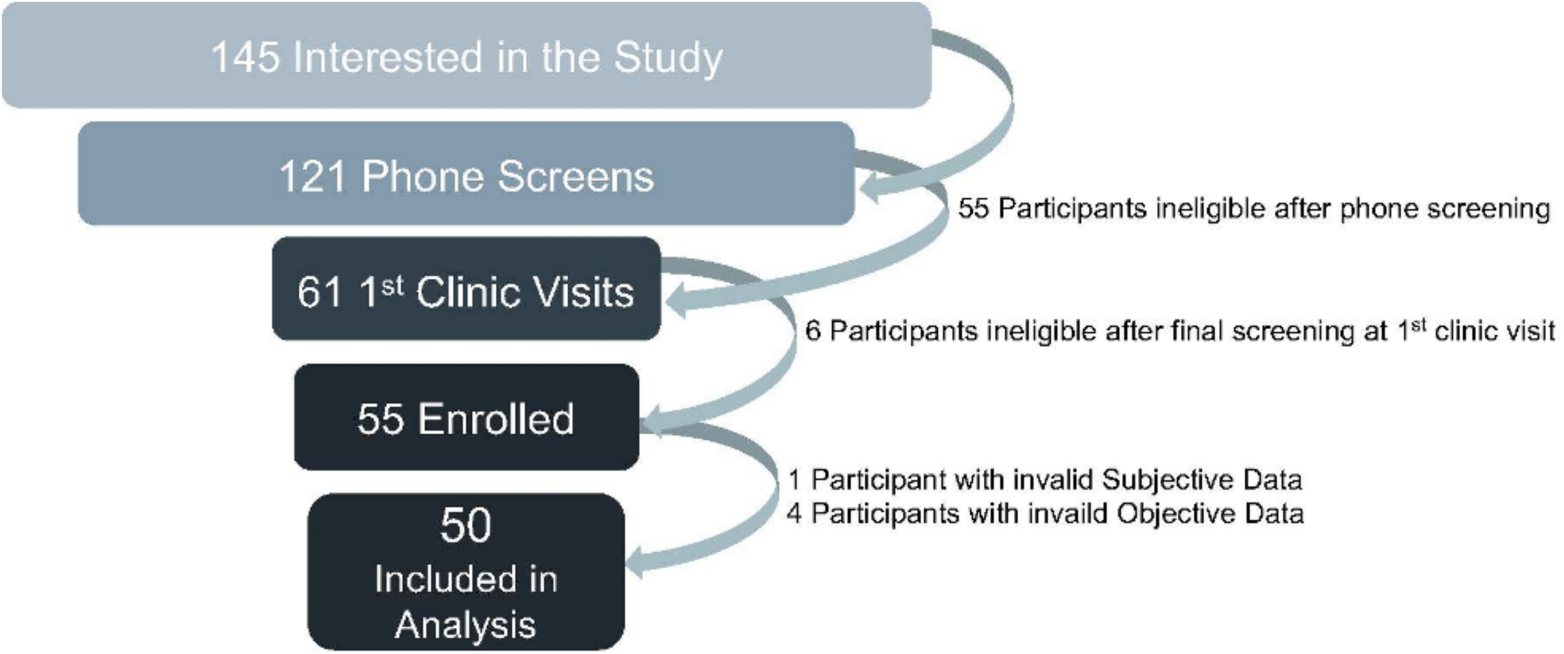
Screening and enrollment.

This study was approved by the Colorado Multiple Institutional Review Board (COMIRB #19-2452). Fifty-five people from the Aurora, Colorado metropolitan area participated in this study. Participants were eligible for the study if they were self-reported light or moderate sleepers between 21 and 65 years old, with difficulty falling or staying asleep primarily due to environmental noise, and who usually slept between 5 and 10 h per night. Potential participants responded to announcements posted online and through flyers to express interest in the study. Coordinators contacted potential participants to confirm eligibility and complete screening. Potential participants were excluded from the study after screening if they had pre-existing sleep disorders, co-occurring conditions, or behavioral factors that were likely to interfere with sleep quality for reasons other than environmental noise, potentially biasing study results. Sleep disorder exclusions were a reported diagnosis of or high risk of pre-existing sleep disorders such as sleep apnea, restless leg syndrome, narcolepsy, or circadian rhythm disorder, assessed by self-report and by response to screening questions used in clinical settings for sleep evaluation, e.g., the STOP-Bang questionnaire for sleep apnea (38). Co-occuring condition exclusions were if potential participants reported significant sleep disturbance due to pain, nocturia, or menopausal symptoms, reported hearing loss or hearing impairment that could interfere with their ability to fully receive the intervention, or were pregnant. Behavioral factor exclusions were if potential participants were likely to be woken during the night due to the presence of young children in the household; if they had irregular schedules, including shift work or delayed sleep phase (usual bedtime after 1 a.m.); or if they reported using stimulants, medications, or products that were likely to affect sleep quality, such as excess alcohol, caffeine, tobacco, or marijuana. Figure 1 depicts a summary of screening and enrollment.

### Procedures

**Figure 2:**
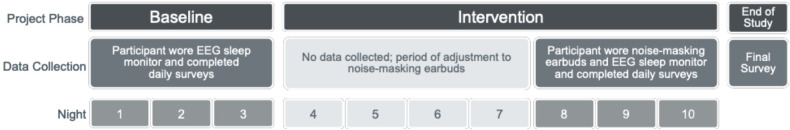
Study timeline.

The study was conducted in three stages over the course of ten consecutive nights, with participants serving as their own controls. The 10-day period was chosen to allow multiple nights for both baseline and intervention data collection (3 nights each, with 2 nights minimum required for inclusion) to reduce the likelihood of data loss leading to subject exclusion due to device issues or operator error, plus a 4-day “washout” period between baseline and intervention to allow participants to acclimate to device use, reducing the chance of poorer sleep quality due to unfamiliar equipment. Figure 2 illustrates the timeline of study activities. Participants were oriented to the study devices during an in-person research visit with the study coordinator, during which they received instruction about device use and had the opportunity to try operating the devices and to ask questions until they were comfortable with their use. During the first three nights (nights 1–3) of the study, participants completed a baseline sleep assessment by responding to daily sleep diary questions in the morning after waking and by wearing a Sleep Profiler™ In-Home EEG Sleep Monitor (Advanced Brain Monitoring, Carlsbad CA) to record physiological signals and measure sleep stages, awakenings, and arousals. The Sleep Profiler has been validated for use in assessing sleep continuity and architecture (39, 40). Over the subsequent four nights (nights 4–7), participants entered an adjustment period where they began wearing the in-ear intervention device and recorded responses to sleep diary questions but did not wear the EEG monitor. Noise-masking sounds, such as nature sounds and tranquil soundtracks, were selected by participants from a list through a dedicated mobile app using an iPod Touch and played in-ear on the intervention device. On the final three nights (nights 8–10), participants wore both the intervention device and the EEG monitor and completed daily sleep diaries. Participants completed a final survey developed specifically for the study at the end of the intervention period. Daily sleep diary questions assessed participants’ perceived success in using the devices, sleep onset latency, level of relaxation, factors interfering with sleep, overall sleep quality, and sleep duration. The final survey assessed participants’ experience and satisfaction with the device and their perceptions of its effectiveness at blocking overall and specific types of environmental noise, improving relaxation, and improving sleep quality. Surveys were self-administered electronically through REDCap (41, 42) electronic data capture tools hosted at the University of Colorado, with reminders and links for survey completion sent to participants daily by email.

### Data analysis

Sample and effect size calculations were conducted *a priori* for a paired *t*-test to evaluate the average change in sleep onset latency with and without the device. Calculations assumed a mean sleep onset latency of 18.6 min without the device and a standard deviation for the difference in sleep onset latency to be 18.5 min. A sample size of 40 was determined to have 80% power at a 0.05 significance level to detect a change in sleep latency of 8.4 min or greater. This change corresponds to the ability to detect Cohen's d effect sizes of 0.454 or greater.

Certified sleep technicians manually scored EEG monitor data according to criteria established by the American Academy of Sleep Medicine (43). A board-certified sleep medicine physician completed final review and interpretation of raw data signals. EEG monitor data were excluded from analysis on a night-by-night basis if either less than four hours of total data was recorded or if more than 50% of the epochs for a single night were deemed invalid due to artifacts (44) that prevented clinical scoring and staging to ensure that sufficient data was available to characterize sleep events for that night. Daily survey data were included in analysis from baseline and intervention periods only, with exclusions made on a night-by-night basis if participants reported that they did not wear the intervention device for the entire night, if the sounds played through the device stopped during the night, or both. Participants were excluded from the analysis as a whole if they had fewer than 2 nights of data in either the baseline or intervention period.

Four primary outcomes were identified *a priori* for statistical inference. Sleep onset latency, wake after sleep onset, and number of awakenings were measured objectively through the EEG monitor. Perceived sleep onset latency was measured subjectively through participant self-report in response to survey items. Mixed-effect linear regression models were fit to each primary outcome to assess the effect of the device, using a random intercept for each participant. Each outcome measure was log-transformed, which improved model fit and supported interpretation on a multiplicative scale. Descriptive statistics were used for exploratory analysis of an additional 36 secondary outcomes, stratified by device use. As these outcomes were measured on discrepant scales, standardized mean differences were calculated and graphed to convey the overall distribution.

As the primary outcomes were specified *a priori* and represented different measurement domains, no adjustments were made for multiple comparisons. To avoid type-1 error inflation, inference was not performed on secondary outcomes. Descriptive differences observed among study participants were reported but are not intended to be representative of broader populations. Since study participants were permitted up to one night of missing data, the *a priori* decision was made to use a chi-square test to assess if missing data was associated with time (e.g., were participants more likely to have missing data on the first night compared to the final night of the 3-night periods). If missingness was observed to be associated with time, a covariate for time was planned for inclusion in the mixed-effect linear regression model analyses.

## Results

Out of 55 participants enrolled in the study, all 55 completed data collection. A total of 50 participants were included in the final analysis (Figure 1), with 5 participants excluded due to insufficient data being collected for analysis (<2 nights in either baseline or intervention periods). EEG monitor data from 279 nights was used for analysis, with a total of 21 nights excluded due to data quality issues. Eleven nights of data were excluded across all survey outcomes, but the distribution of which nights were excluded for which participant varied based on participant response patterns. Some survey items had additional missing nights if participants omitted responding for that item. However, no differential patterns were observed in the distribution across nights.

Most participants were white (64%) and employed full time (86%). Table 1 further describes population demographics among study participants.

**Table 1:** Study population characteristics.

	Overall (*n* = 50)
Age	
21–25 years old	8 (16.0%)
26–35 years old	14 (28.0%)
36–45 years old	16 (32.0%)
46–55 years old	10 (20.0%)
56–65 years old	2 (4.0%)
Ethnicity	
Asian or Pacific Islander	3 (6.0%)
Black/African American	1 (2.0%)
Hispanic/Latino or Latina	11 (22.0%)
Other/Multiple	3 (6.0%)
White/Caucasian	32 (64.0%)
Employment status	
Full-time	43 (86.0%)
Full-time student	2 (4.0%)
Not employed, not looking for work	1 (2.0%)
Part-time	4 (8.0%)
Total annual household income before taxes	
<$25,000	2 (4.0%)
$25,000-$49,999	7 (14.0%)
$50,000-$74,999	7 (14.0%)
$75,000-$99,999	4 (8.0%)
$100,000-$124,999	5 (10.0%)
$125,000-$149,999	11 (22.0%)
$150,000-$174,999	3 (6.0%)
$175,000-$199,999	1 (2.0%)
$200,000 or more	2 (4.0%)
Which best describes the building you live in?	
One-family house detached from others	24 (48.0%)
One-family house attached to one or more buildings	9 (18.0%)
Building with 2–3 apartments	3 (6.0%)
Building with 4 or more apartments	14 (28.0%)
Dorm	0 (0%)
Other	0 (0%)
Would you describe where you live as:	
Urban	24 (48.0%)
Suburban	26 (52.0%)
Rural	0 (0%)
Other	0 (0%)

Use of the device was associated with statistically significant reductions in both perceived sleep onset latency and sleep onset latency measured through the EEG monitor. Perceived sleep latency was 32.5% faster (95% C.I. = 22.3%–41.4%; *p* < 0.001) and measured sleep onset latency was 13.3% faster (95% C.I. = 1.4%–23.8%; *p* = 0.03) as compared to baseline. There were no statistically significant differences found in wake after sleep onset (5.0% less; C.I. = 6.7% more to 15.4% less; *p* = 0.385) and number of awakenings (3.2% less; C.I. = 3.6% more to 9.6% less; *p* = 0.349) when using the intervention device as compared to baseline. Table 2 shows the results for the four primary outcomes in more detail.

**Table 2:** Primary outcomes stratified by baseline vs. intervention time periods.

Outcome	Baseline mean (95% C.I.)	Device mean (95% C.I.)	Mean difference	*p*-value
Sleep onset latency, minutes	17.0 (13.7, 20.3)	14.3 (11.4, 17.1)	2.7 (2.3, 3.2)	*p* = 0.030
Wake after sleep onset, minutes	48.3 (41.2, 55.4)	46.3 (39.8, 52.8)	2.0 (1.4, 2.6)	*p* = 0.385
Number of awakenings, *n*	28.0 (25.6, 30.4)	27.1 (24.8, 29.4)	0.9 (0.8, 1.0)	*p* = 0.349
Perceived sleep onset latency, minutes	23.8 (20.4, 27.2)	16.3 (13.7, 19.0)	7.5 (6.7, 8.2)	*p* < 0.001

**Figure 3:**
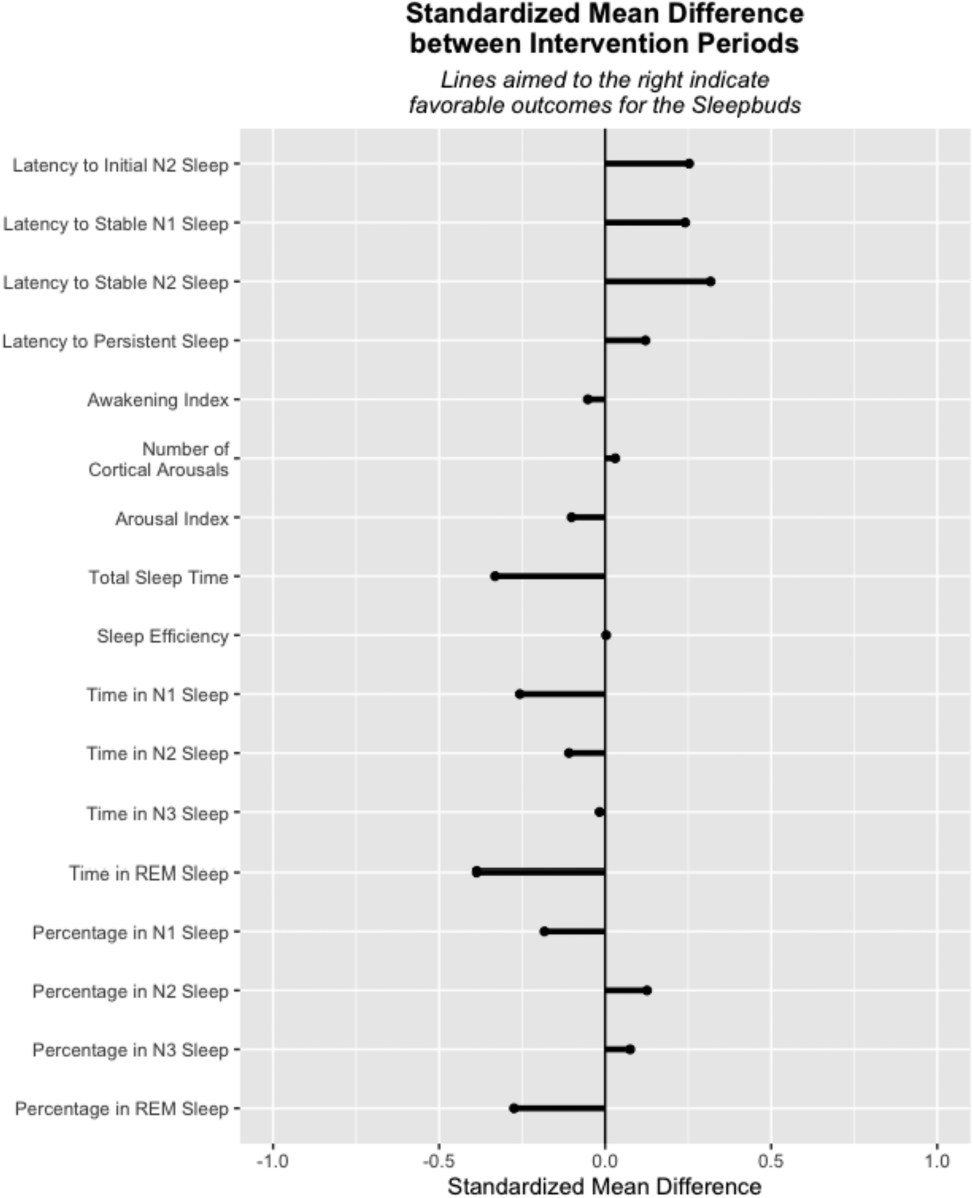
Standardized mean differences for exploratory outcomes from the sleep profiler™.

**Figure 4:**
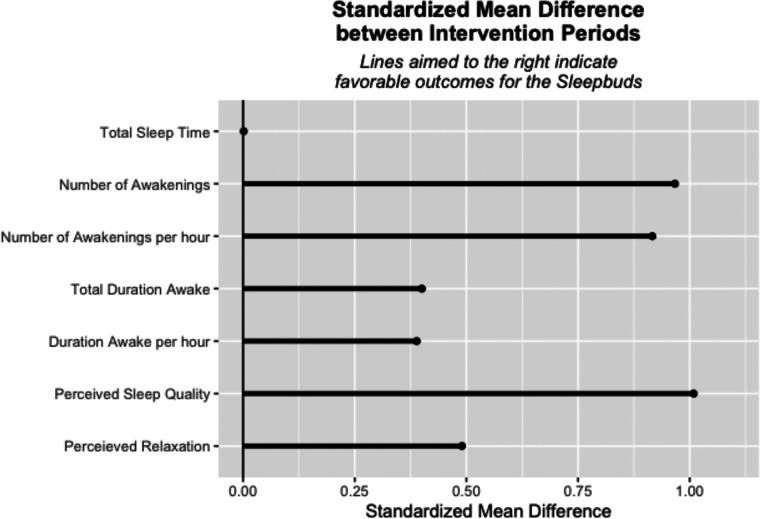
Standardized mean differences for exploratory outcomes from daily surveys.

Seventeen secondary outcomes objectively measured using the EEG monitor are summarized in Figure 3 as standardized effect sizes with a common directionality. These outcomes included latency to and time in different stages of sleep, were neither consistently favorable nor unfavorable to the intervention, and in aggregate appear evenly distributed around no difference of intervention. Nineteen subjectively measured secondary outcomes are reported, seven from responses to daily surveys and 12 from responses to the post-intervention survey. These outcomes included perceptions of device impact on sleep and sleep quality and device effectiveness at blocking different types of environmental noise. Figure 4 depicts the standardized effect sizes with a common directionality for the seven daily surveys. In contrast to the objectively measured secondary outcomes, all of the subjective daily measures show pre/post descriptive differences favorable to the intervention. Table 3 depicts objective secondary outcome results from the EEG monitor and subjective secondary outcomes from the daily surveys. Table 4 depicts secondary outcome results from the final survey. Study participants broadly reported improved sleep quality when wearing the study device, across all 17 subjectively reported secondary outcomes.

**Table 3A:** Secondary outcomes, objective (sleep profiler™).

Outcome	Baseline mean (95% C.I.)	Sleepbuds^TM^ mean (95% C.I.)
Latency to initial N2 sleep, minutes	19.3 (15.9, 22.6)	16.4 (13.4, 19.4)
Latency to stable N1 sleep, minutes	17.1 (13.9, 20.4)	14.4 (11.5, 17.4)
Latency to stable N2 sleep, minutes	20.9 (17.2, 24.7)	17.0 (13.9, 20.1)
Latency to persistent sleep, minutes	21.3 (17.2, 25.3)	19.5 (15.3, 23.6)
Awakening index,%	3.6 (3.4, 3.9)	3.7 (3.4, 4.0)
Number of cortical arousals, n	156.2 (136.7, 175.8)	154.2 (137.5, 171.0)
Arousal index,%	20.2 (17.9, 22.4)	21.0 (19.0, 23.0)
Total sleep time, minutes	392.2 (377.2, 407.3)	374.2 (359.2, 389.3)
Sleep efficiency,%	51.2 (50.2, 52.2)	51.3 (50.1, 52.4)
Time in N1 sleep, minutes	16.3 (14.2, 18.5)	14.4 (12.3, 16.4)
Time in N2 sleep, minutes	213.4 (201.1, 225.8)	208.4 (195.5, 221.4)
Time in N3 sleep, minutes	65.8 (53.5, 78.1)	65.1 (54.2, 76.0)
Time in REM sleep, minutes	96.6 (89.6, 103.7)	86.3 (78.5, 94.0)
Percentage in N1 Sleep,%	2.1 (1.9, 2.4)	2.0 (1.7, 2.2)
Percentage in N2 Sleep,%	27.9 (26.6, 29.1)	28.5 (27.0, 30.0)
Percentage in N3 Sleep,%	8.7 (7.2, 10.3)	9.1 (7.6, 10.7)
Percentage in REM sleep,%	12.6 (11.8, 13.3)	11.7 (10.7, 12.7)

**Table 3B:** Secondary outcomes, daily surveys.

Outcome	Baseline mean (95% C.I.)	Sleepbuds^TM^ mean (95% C.I.)
Total sleep time, hours	7.2 (7.0, 7.4)	7.2 (7.0, 7.4)
Number of awakenings, *n*	3.1 (2.7, 3.5)	1.9 (1.6, 2.2)
Number of awakenings per hour, *n*	0.4 (0.4, 0.5)	0.3 (0.2, 0.3)
Total duration awake, minutes	20.6 (15.3, 26.0)	14.3 (11.2, 17.4)
Duration awake per hour, minutes	3.1 (2.3, 3.9)	2.2 (1.7, 2.6)
Perceived sleep quality, 9-point scale (lower is better)	4.8 (4.5, 5.1)	3.6 (3.3, 3.9)
Perceived relaxation, 9-point scale (lower is better)	4.3 (4.0, 4.6)	3.8 (3.5, 4.1)

**Table 4:** Secondary outcomes, final survey.

Effect of device on the ability to fall asleep	
Negative effect	3 (6.0%)
No effect	4 (8.0%)
Positive effect	43 (86.0%)
Effect of device on the ability to stay asleep	
Negative effect	7 (14.0%)
No effect	5 (10.0%)
Positive effect	38 (76.0%)
Effect of device on the overall quality of sleep	
Negative effect	5 (10.0%)
No effect	4 (8.0%)
Positive effect	41 (82.0%)
Effect of device on blocking overall sounds	
No effect	0 (0%)
Positive effect	50 (100%)
Effect of device on blocking snoring	
No effect	0 (0%)
Positive effect	39 (100%)
Not applicable	11
Effect of device on blocking pets	
No effect	1 (3.4%)
Positive effect	28 (96.6%)
Not applicable	21
Effect of device on blocking children	
No effect	0 (0%)
Positive effect	18 (100%)
Not applicable	32
Effect of device on blocking inside house noises	
No effect	1 (2.3%)
Positive effect	43 (97.7%)
Not applicable	6
Effect of device on blocking road traffic	
No effect	0 (0%)
Positive effect	44 (100%)
Not applicable	6
Effect of device on blocking air traffic	
No effect	0 (0%)
Positive effect	33 (100%)
Not applicable	17
Effect of device on blocking crowds/people	
No effect	0 (0%)
Positive effect	30 (100%)
Not applicable	20
Likely to continue device use afterwards from 0 (not likely at all) to 10 (extremely likely)	
Mean (SD)	6.98 (2.97)
Median [IQR]	8 (5, 10)

Categorical responses for the effect of Bose noise masking sleepbuds^TM^ are summarized and provided as the number of respondents (%). The likelihood to continue use is summarized from an integer scale (0–10) using the mean (standard deviation) and median [interquartile range (IQR)].

## Discussion

The aims of this study were to assess the impact of noise-masking earbuds on improving sleep quality, both as subjectively perceived and as objectively measured through EEG data, for people experiencing issues with sleep initiation and sleep maintenance due to environmental noise at home.

Study participants consistently reported better sleep quality when using the in-ear noise-masking device across primary and secondary subjective outcomes. Sleep onset latency measured both objectively and subjectively showed a statistically significant effect in favor of the device, ranging in improvement from 13.3% to 32.5% respectively. These findings are not only consistent with results from other studies using similar in-ear noise masking devices (35, 36), but are also consistent with results previously observed in studies among adults in hospital settings using non-pharmacological interventions (45–47). Noise masking solutions in particular have been shown in clinical trials to improve sleep outcomes between 22.9% and 37.5% (48, 49).

Other objective measurements of sleep quality did not show a consistent statistically significant effect (for primary outcomes of WASO and number of awakenings) or descriptive difference (for secondary outcomes) favorable to the intervention. Such variation between results has previously been observed in systematic reviews of sleep research involving non-pharmacological interventions (50). One possible cause of the discrepancy for this study in particular, supported by research assessing different elements of sleep quality via both objective and subjective means, is the existence of discordance between the elements of sleep quality which sleepers are truly able to subjectively assess vs. what can be reliably detected using EEG data and polysomnography (51–53). Future studies may benefit from exploring additional and alternate means for objective measurement, such as wrist actigraphy or smartwatch assessment and using validated instruments for self-reported measures that could facilitate more direct comparison, in order to address this issue.

One limitation of this study is the possibility for participants to become acclimated to wearing the EEG monitor over time, potentially leading to better sleep during the intervention period vs. baseline. We addressed this limitation by including a 4-day period between baseline and intervention periods to allow for wash-out and minimize this potential effect. Another limitation is the predominantly affluent, white, and urban demographics of the study population, as this population may reside in locations with differing levels of impact due to environmental noise than lower-income or minority persons or rural residents. More research is needed with diverse populations to better understand the potential use of noise-masking earbuds with other groups and in additional environments. Future longer-term studies should address these limitations and considerations.

This study is innovative in using electroencephalographic measurement of sleep stages under real-world conditions in a home setting rather than in a sleep laboratory or other controlled environment, allowing for comparison to self-reported measures more commonly used in home environments. Future studies might build upon this approach by using similar EEG-based measurements to directly compare with commonly used methods for home-based data collection such as wrist actigraphy and smartwatch wearables. Additionally, while the pre/post design was appropriate for this initial efficacy study, future studies may also benefit from the inclusion of a separate control group to better support conclusions generalizable to a broader population.

It should be noted that although study participants self-identified as being light or moderate sleepers and also identified that they had snoring partners or environmental noise which they found disruptive, this group demonstrated normal sleep onset latency times. If the findings from this study are sustained at the population level, this represents a significant potential public health benefit for people living in noisy environments who are not receiving care for sleep-related conditions. It would also be beneficial to evaluate whether similar improvement in both perceived and objective sleep onset latency is seen among patients with clinically diagnosed insomnia, who experience prolonged sleep onset latency. If so, the potential impact on clinical practice and benefit to patients may be significant, especially if easily accessible, commercially available non-pharmacological noise-masking solutions can be used to replace sedative medications without reducing effectiveness.

## Conclusion

Results from this pilot study suggest noise-masking earbuds may be used in a home setting to improve sleep onset latency and users’ perception of sleep quality more generally. When considering practical applications of this research, this study supports the conclusion that as a digital health wellness device, noise-masking earbuds could be used as part of a holistic, comprehensive approach to good sleep management and improved sleep hygiene without the potential side effects attributable to pharmaceutical interventions. In particular, the device showed great promise for use in noisy environments such as in high-density urban settings. Given the importance of good sleep to optimal health, employing a non-invasive technological solution to improve sleep quality represents a potential low-cost, accessible, and low-risk advancement for public health.

## Data Availability

The datasets presented in this article are not readily available to ensure participant privacy is protected. Requests to access a manually deidentified version of the datasets should be directed to the corresponding author.

## References

[1] Watson NF Badr MS Belenky G Bliwise DL Buxton OM Buysse D Joint consensus statement of the American academy of sleep medicine and sleep research society on the recommended amount of sleep for a healthy adult: methodology and discussion. Sleep. (2015) 38(8):1161–83. 10.5665/sleep.4886 26194576 PMC4507722

[2] Bertisch SM Pollock BD Mittleman MA Buysse DJ Bazzano LA Gottlieb DJ Insomnia with objective short sleep duration and risk of incident cardiovascular disease and all-cause mortality: sleep heart health study. Sleep. (2018) 41(6):1–9. 10.1093/sleep/zsy047 PMC5995202 29522193

[3] Cappuccio FP D’Elia L Strazzullo P Miller MA. Sleep duration and all-cause mortality: a systematic review and meta-analysis of prospective studies. Sleep. (2010) 33(5):585–92. 10.1093/sleep/33.5.585 20469800 PMC2864873

[4] Kwok CS Kontopantelis E Kuligowski G Gray M Muhyaldeen A Gale CP Self-reported sleep duration and quality and cardiovascular disease and mortality: a dose-response meta-analysis. J Am Heart Assoc. (2018) 7(15):e008552. 10.1161/JAHA.118.008552 30371228 PMC6201443

[5] Yin J Jin X Shan Z Li S Huang H Li P Relationship of sleep duration with all-cause mortality and cardiovascular events: a systematic review and dose-response meta-analysis of prospective cohort studies. J Am Heart Assoc. (2017) 6(9):1–15. 10.1161/JAHA.117.005947 PMC5634263 28889101

[6] Hsieh CG Martin JL. Short sleep, insomnia, and cardiovascular disease. Curr Sleep Med Rep. 2019;5(4):234–42. 10.1007/s40675-019-00157-8 33344144 PMC7747467

[7] Khan MS Aouad R. The effects of insomnia and sleep loss on cardiovascular disease. Sleep Med Clin. (2017) 12(2):167–77. 10.1016/j.jsmc.2017.01.005 28477772

[8] Kim CW Chang Y Kang JG Ryu S. Changes in sleep duration and subsequent risk of hypertension in healthy adults. Sleep. (2018) 41(11). 10.1093/sleep/zsy159 30137630

[9] Cappuccio FP D’Elia L Strazzullo P Miller MA. Quantity and quality of sleep and incidence of type 2 diabetes: a systematic review and meta-analysis. Diabetes Care. (2010) 33(2):414–20. 10.2337/dc09-1124 19910503 PMC2809295

[10] Grandner MA Seixas A Shetty S Shenoy S. Sleep duration and diabetes risk: population trends and potential mechanisms. Curr Diab Rep. (2016) 16(11):106. 10.1007/s11892-016-0805-8 27664039 PMC5070477

[11] Cappuccio FP Miller MA. Sleep and cardio-metabolic disease. Curr Cardiol Rep. (2017) 19(11):110. 10.1007/s11886-017-0916-0 28929340 PMC5605599

[12] Dashti HS Scheer FA Jacques PF Lamon-Fava S Ordovás JM. Short sleep duration and dietary intake: epidemiologic evidence, mechanisms, and health implications. Adv Nutr. (2015) 6(6):648–59. 10.3945/an.115.008623 26567190 PMC4642416

[13] Zimberg IZ Dâmaso A Del Re M Carneiro AM de Sá Souza H de Lira FS Short sleep duration and obesity: mechanisms and future perspectives. Cell Biochem Funct. (2012) 30(6):524–9. 10.1002/cbf.2832 22473743

[14] Basner M McGuire S. WHO environmental noise guidelines for the European region: a systematic review on environmental noise and effects on sleep. Int J Environ Res Public Health. (2018) 15(3):519. 10.3390/ijerph15030519 29538344 PMC5877064

[15] Li L Wu C Gan Y Qu X Lu Z. Insomnia and the risk of depression: a meta-analysis of prospective cohort studies. BMC Psychiatry. (2016) 16(1):375. 10.1186/s12888-016-1075-3 27816065 PMC5097837

[16] Zhai L Zhang H Zhang D. Sleep duration and depression among adults: a meta-analysis of prospective studies. Depress Anxiety. (2015) 32(9):664–70. 10.1002/da.22386 26047492

[17] Edinger JD Bonnet MH Bootzin RR Doghramji K Dorsey CM Espie CA Derivation of research diagnostic criteria for insomnia: report of an American academy of sleep medicine work group. Sleep. (2004) 27(8):1567–96. 10.1093/sleep/27.8.1567 15683149

[18] Hohagen F Käppler C Schramm E Riemann D Weyerer S Berger M. Sleep onset insomnia, sleep maintaining insomnia and insomnia with early morning awakening–temporal stability of subtypes in a longitudinal study on general practice attenders. Sleep. (1994) 17(6):551–4. 7809569

[19] Hume KI Brink M Basner M. Effects of environmental noise on sleep. Noise Health. (2012) 14(61):297–302. 10.4103/1463-1741.104897 23257581

[20] Kawada T. Noise and health—sleep disturbance in adults. J Occup Health. (2011) 53(6):413–6. 10.1539/joh.11-0071-RA 21952296

[21] Röösli M Brink M Rudzik F Cajochen C Ragettli MS Flückiger B Associations of various nighttime noise exposure indicators with objective sleep efficiency and self-reported sleep quality: a field study. Int J Environ Res Public Health. (2019) 16(20):1–13. 10.3390/ijerph16203790 PMC6843841 31600891

[22] Halperin D. Environmental noise and sleep disturbances: a threat to health? Sleep Sci. (2014) 7(4):209–12. 10.1016/j.slsci.2014.11.003 26483931 PMC4608916

[23] Münzel T Schmidt FP Steven S Herzog J Daiber A Sørensen M. Environmental noise and the cardiovascular system. J Am Coll Cardiol. (2018) 71(6):688–97. 10.1016/j.jacc.2017.12.015 29420965

[24] Basner M Babisch W Davis A Brink M Clark C Janssen S Auditory and non-auditory effects of noise on health. Lancet. (2014) 383(9925):1325–32. 10.1016/S0140-6736(13)61613-X 24183105 PMC3988259

[25] Hahad O Prochaska JH Daiber A Muenzel T. Environmental noise-induced effects on stress hormones, oxidative stress, and vascular dysfunction: key factors in the relationship between cerebrocardiovascular and psychological disorders. Oxid Med Cell Longev. (2019) 2019:4623109. 10.1155/2019/4623109 31814877 PMC6878772

[26] APHA. Noise as a Public Health Hazard. Washington, DC: APHA (2021). Available online at: https://apha.org/policies-and-advocacy/public-health-policy-statements/policy-database/2022/01/07/noise-as-a-public-health-hazard.

[27] Gerrit JDN Tiemens BG Kloos MW Hutschemaekers GJ. Review of systematic reviews about the efficacy of non-pharmacological interventions to improve sleep quality in insomnia. Int J Evid Based Healthc. (2009) 7(4):233–42. 10.1111/j.1744-1609.2009.00142.x 21631864

[28] Feng F Zhang Y Hou J Cai J Jiang Q Li X Can music improve sleep quality in adults with primary insomnia? A systematic review and network meta-analysis. Int J Nurs Stud. (2018) 77:189–96. 10.1016/j.ijnurstu.2017.10.011 29100201

[29] Murawski B Wade L Plotnikoff RC Lubans DR Duncan MJ. A systematic review and meta-analysis of cognitive and behavioral interventions to improve sleep health in adults without sleep disorders. Sleep Med Rev. (2018) 40:160–9. 10.1016/j.smrv.2017.12.003 29397329

[30] Rusch HL Rosario M Levison LM Olivera A Livingston WS Wu T The effect of mindfulness meditation on sleep quality: a systematic review and meta-analysis of randomized controlled trials. Ann N Y Acad Sci. (2019) 1445:5–16. 10.1111/nyas.13996 30575050 PMC6557693

[31] Trauer JM Qian MY Doyle JS Rajaratnam SM Cunnington D. Cognitive behavioral therapy for chronic insomnia. Ann Intern Med. (2015) 163(3):191–204. 10.7326/M14-2841 26054060

[32] Wen X Yang H Wang J. Application of noise reduction earplugs in patients undergoing total knee arthroplasty: a retrospective study. Noise Health. (2024) 26(120):19–24. 10.4103/nah.nah_88_23 38570306 PMC11141701

[33] Huang D Li Y Ye J Liu C Shen D Lv Y. Different nursing interventions on sleep quality among critically ill patients: a systematic review and network meta-analysis. Medicine (Baltimore). (2023) 102(52):e36298. 10.1097/MD.0000000000036298 38206715 PMC10754598

[34] Tian M Gu X. Effect of white noise intervention combined with multi-dimensional nursing mode on sleep quality and incidence of nosocomial infection in patients undergoing hip replacement. Noise Health. (2023) 25(119):220–5. 10.4103/nah.nah_32_23 38358237 PMC10849012

[35] Haller HC Moore SL Green KK Johnson RL Sammel MD Epperson CN Harnessing technology to improve sleep in frontline healthcare workers: a pilot study of electronic noise-masking earbuds on subjective and objective sleep measures. Sci Prog. (2024) 107(2):368504241242276. 10.1177/00368504241242276 38614463 PMC11016237

[36] Duggan NM Hasdianda MA Baker O Jambaulikar G Goldsmith AJ Condella A The effect of noise-masking earbuds (SleepBuds) on reported sleep quality and tension in health care shift workers: prospective single-subject design study. JMIR Form Res. (2022) 6(3):e28353. 10.2196/28353 35315781 PMC8984824

[37] Buysse DJ Reynolds CF 3rd Monk TH Berman SR Kupfer DJ. The Pittsburgh sleep quality index: a new instrument for psychiatric practice and research. Psychiatry Res. (1989) 28(2):193–213. 10.1016/0165-1781(89)90047-4 2748771

[38] Chung F Abdullah HR Liao P. STOP-bang questionnaire: a practical approach to screen for obstructive sleep apnea. Chest. (2016) 149(3):631–8. 10.1378/chest.15-0903 26378880

[39] Levendowski DJ Popovic D Berka C Westbrook PR. Retrospective cross-validation of automated sleep staging using electroocular recording in patients with and without sleep disordered breathing. Int Arch Med. (2012) 5(1):21. 10.1186/1755-7682-5-21 22726270 PMC3436769

[40] Finan PH Richards JM Gamaldo CE Han D Leoutsakos JM Salas R Validation of a wireless, self-application, ambulatory electroencephalographic sleep monitoring device in healthy volunteers. J Clin Sleep Med. (2016) 12(11):1443–51. 10.5664/jcsm.6262 27707438 PMC5078698

[41] Harris P Taylor R Minor B Elliott V Fernandez M O’Neal L The REDCap consortium: building an international community of software partners. J Biomed Inform. (2019) 95:103208. 10.1016/j.jbi.2019.103208 31078660 PMC7254481

[42] Harris P Taylor R Thielke R Payne J Gonzalez N Conde J. Research electronic data capture (REDCap)—a metadata-driven methodology and workflow process for providing translational research informatics support. J Biomed Inform. (2009) 42(2):377–81. 10.1016/j.jbi.2008.08.010 18929686 PMC2700030

[43] Berry RB Brooks R Gamaldo C Harding SM Lloyd RM Quan SF AASM scoring manual updates for 2017 (version 2.4). J Clin Sleep Med. (2017) 13(5):665–6. 10.5664/jcsm.6576 28416048 PMC5406946

[44] Campbell IG. EEG recording and analysis for sleep research. Curr Protoc Neurosci. (2009) Chapter 10:Unit10.2. 10.1002/0471142301.ns1002s49 19802813 PMC2824445

[45] Hu RF Jiang XY Hegadoren KM Zhang YH. Effects of earplugs and eye masks combined with relaxing music on sleep, melatonin and cortisol levels in ICU patients: a randomized controlled trial. Crit Care. (2015) 19(1):115. 10.1186/s13054-015-0855-3 25881268 PMC4391192

[46] Afshar PF Bahramnezhad F Asgari P Shiri M. Effect of white noise on sleep in patients admitted to a coronary care. J Caring Sci. (2016) 5(2):103–9. 10.15171/jcs.2016.011 27354974 PMC4923834

[47] Obanor OO McBroom MM Elia JM Ahmed F Sasaki JD Murphy KM The impact of earplugs and eye masks on sleep quality in surgical ICU patients at risk for frequent awakenings. Crit Care Med. (2021) 49(9):e822–32. 10.1097/CCM.0000000000005031 33870919

[48] Williamson JW. The effects of ocean sounds on sleep after coronary artery bypass graft surgery. Am J Crit Care. (1992) 1(1):91–7. 10.4037/ajcc1992.1.1.91 1307884

[49] Xie H Kang J Mills GH. Clinical review: the impact of noise on patients’ sleep and the effectiveness of noise reduction strategies in intensive care units. Crit Care. (2009) 13(2):208. 10.1186/cc7154 19344486 PMC2689451

[50] Hu RF Jiang XY Chen J Zeng Z Chen XY Li Y Non-pharmacological interventions for sleep promotion in the intensive care unit. Cochrane Database Syst Rev. (2015) 2015(10):Cd008808. 10.1002/14651858.CD008808.pub2 26439374 PMC6517220

[51] O’Donnell D Silva EJ Münch M Ronda JM Wang W Duffy JF. Comparison of subjective and objective assessments of sleep in healthy older subjects without sleep complaints. J Sleep Res. (2009) 18(2):254–63. 10.1111/j.1365-2869.2008.00719.x 19645969 PMC2975570

[52] Baker FC Maloney S Driver HS. A comparison of subjective estimates of sleep with objective polysomnographic data in healthy men and women. J Psychosom Res. (1999) 47(4):335–41. 10.1016/S0022-3999(99)00017-3 10616227

[53] Cudney LE Frey BN McCabe RE Green SM. Investigating the relationship between objective measures of sleep and self-report sleep quality in healthy adults: a review. J Clin Sleep Med. (2022) 18(3):927–36. 10.5664/jcsm.9708 34609276 PMC8883085

